# HIV-1 Envelope Glycosylation and the Signal Peptide

**DOI:** 10.3390/vaccines9020176

**Published:** 2021-02-19

**Authors:** Gregory S. Lambert, Chitra Upadhyay

**Affiliations:** Division of Infectious Diseases, Department of Medicine, Icahn School of Medicine at Mount Sinai, 1 Gustave L. Levy Place, New York, NY 10029, USA; gregory.lambert@mssm.edu

**Keywords:** HIV-1, HIV envelope, glycosylation, signal peptide, PNGS, broadly neutralizing antibodies, vaccine

## Abstract

The RV144 trial represents the only vaccine trial to demonstrate any protective effect against HIV-1 infection. While the reason(s) for this protection are still being evaluated, it serves as justification for widespread efforts aimed at developing new, more effective HIV-1 vaccines. Advances in our knowledge of HIV-1 immunogens and host antibody responses to these immunogens are crucial to informing vaccine design. While the envelope (Env) protein is the only viral protein present on the surface of virions, it exists in a complex trimeric conformation and is decorated with an array of variable N-linked glycans, making it an important but difficult target for vaccine design. Thus far, efforts to elicit a protective humoral immune response using structural mimics of native Env trimers have been unsuccessful. Notably, the aforementioned N-linked glycans serve as a component of many of the epitopes crucial for the induction of potentially protective broadly neutralizing antibodies (bnAbs). Thus, a greater understanding of Env structural determinants, most critically Env glycosylation, will no doubt be of importance in generating effective immunogens. Recent studies have identified the HIV-1 Env signal peptide (SP) as an important contributor to Env glycosylation. Further investigation into the mechanisms by which the SP directs glycosylation will be important, both in the context of understanding HIV-1 biology and in order to inform HIV-1 vaccine design.

## 1. Introduction

Infection of CD4+ T cells by the human immunodeficiency virus type 1 (HIV-1) leads to a drastic decrease in their number and causes acquired immune deficiency syndrome (AIDS), which can eventually lead to death. Treatment of individuals infected with HIV-1 consists of the administration of highly effective antiretroviral medications, which allow patients to live relatively normal lives assuming that treatment regimens are adhered to [1,2,3]. However, resistance to these medications is of some concern, and therefore additional treatment approaches are of importance [4]. In the absence of a cure, prevention of new infections via prophylactic vaccination is likely to have a more widespread and lasting effect and is therefore the main focus of HIV-1 research [5].

Due to the high selective pressure induced by the immune system and the high mutation rate of HIV-1, chronic infection often consists of multiple viral quasispecies [6,7]. The immune response of an HIV-1 infected individual therefore needs to effectively prevent multiple viral quasispecies from infecting CD4+ T cells. This can be achieved via the induction of a broad milieu of antibodies (Abs) with varying specificities that act in concert to prevent infection but is ideally mediated via antibodies capable of binding epitopes that are conserved between many HIV-1 strains [8]. These antibodies are known as broadly neutralizing antibodies (bnAbs) and attempts to induce their production via vaccination have been a focal point of HIV-1 vaccine design.

The trimeric envelope (Env) that is responsible for initiating HIV-1 infection is the sole target for the induction of neutralizing antibodies (nAbs). Much effort has been expended to create recombinant Env trimers that share great structural similarity to native Env trimers (termed SOSIP, uncleaved prefusion optimized (UFO), single-chain (SC), and native-flexible-linker (NFL), among others), with the hope of inducing bnAbs. However, none have thus far succeeded in this aim. These efforts are no doubt a crucial step in immunogen design and have been reviewed elsewhere [9,10]. Complicating these efforts is the existence of a wide array of N-linked glycan structures on the Env surface that modulate its interaction with the host immune response and whose composition is dependent on a number of variable viral and host factors. One such viral factor, the Env signal peptide (SP), has recently been shown to greatly influence the composition of N-glycans on Env [11]. Notably, glycosylation of the HIV-1 Env can modulate the antibody response. This review highlights challenges in HIV-1 vaccine design related to Env glycosylation, with emphasis on the contribution of the Env SP in directing said glycosylation.

## 2. HIV-1 Envelope Structure and Immunogenicity

**Figure 1:**
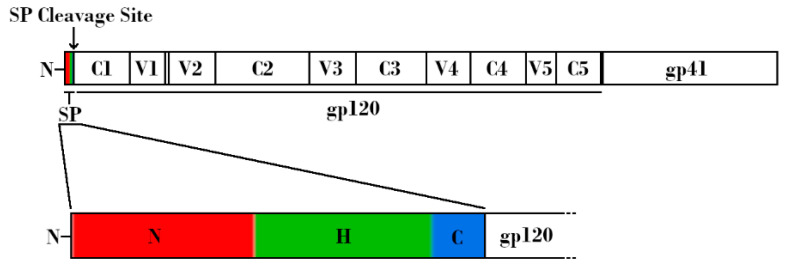
Schematic linear representation of the nascent HIV-1 Envelope (Env) protein attached to the HIV-1 signal peptide (SP). (Top) Regions corresponding to SP, glycoprotein 120 (gp120), and glycoprotein 41 (gp41) are indicated. Variable and constant regions of gp120 are indicated by V1-V5 and C1-C5, respectively. SP cleavage site is indicated by an arrow. (Bottom) Expanded schematic representation of the HIV-1 SP. The N-terminal hydrophilic positively charged region is shown in red, the central hydrophobic region is shown in green, and the slightly polar C-terminal region is shown in blue.

**Figure 2:**
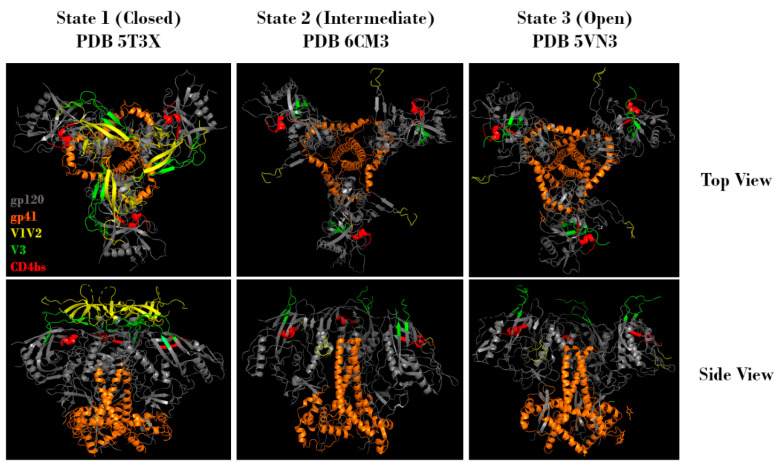
Representative top and side views of Env trimers in state 1 (closed), state 2 (intermediate), and state 3 (open) conformations. Regions corresponding to gp120 (grey), gp41 (orange), V1V2 (yellow), V3 (green), and the CD4 binding site (red) are indicated. Protein Data Bank identifier (PDB ID) numbers are also indicated. Structures adapted from Gristick et al., 2016 [26], Bjorkman et al., 2018 [27], and Ozorowski et al., 2017 [28].

In order to understand how Env glycosylation complicates efforts to create effective Env-based immunogens, it is important to understand the structure, function, and inherent immune evasion properties of Env. HIV-1 Env exists as a trimeric spike on the viral surface, consisting of three heterodimers. These heterodimers arise from furin-mediated cleavage of glycoprotein 160 (gp160) proteins, resulting in a glycoprotein 120 (gp120) non-covalently linked to a glycoprotein 41 (gp41) (Figure 1, top). The gp120 subunit can be further subdivided into five conserved regions (C1-C5) and five hypervariable regions (V1-V5) [12,13,14,15]. Binding to host cells is facilitated by the gp120 subunit, which contains both the CD4 and co-receptor (either CCR5 or CXCR4) binding sites. Upon binding, Env undergoes conformational changes that allow gp41 to mediate fusion of the viral membrane with the host cell membrane, leading to infection [6,13,16,17]. Env is a dynamic molecule that exists in one of three conformations at any given time: a metastable closed conformation (state 1), a partially open intermediate conformation (state 2), and an open conformation (state 3) (Figure 2). Env transitions between these three conformations, but the majority of known bnAbs, isolated from HIV-1 infected individuals, target Env in state 1. Of note, the current recombinant Env trimers present Env in a stabilized version of state 2 [18]. In state 2 or 3, the variable loops V1V2 and V3 are more available for immune recognition. This functionally means that the initial antibody response is often specific to the infecting strain, and that escape mutations are common. Uncleaved gp160 monomers, as well as gp41 stumps lacking an associated gp120, also exist on the viral surface. These nonfunctional proteins display epitopes that may lead to the generation of non-neutralizing antibodies (nnAbs). Combined with the relatively low number of functional Env trimers, these mechanisms serve to effectively divert the host immune response [19,20,21,22,23,24,25].

## 3. HIV-1 Env N-linked Glycosylation

The existence of a wide array of N-glycans on the Env surface adds an additional layer of complexity to the already highly variable Env. Each Env of the trimer contains between 18 and 33 potential N-linked glycosylation sites (PNGSs), whose occupancy by glycans can account for up to half of the trimer’s total mass and cover up to 50–70% of the Env surface [7,29,30,31,32]. In this way, these poorly immunogenic glycans shield underlying protein residues from immune recognition. The occupancy of any particular PNGS is variable, with most being less than 90% conserved between HIV-1 strains [33,34]. Most PNGSs exist on gp120, but ~4–5 can be found on gp41 as well [33].

**Figure 3:**
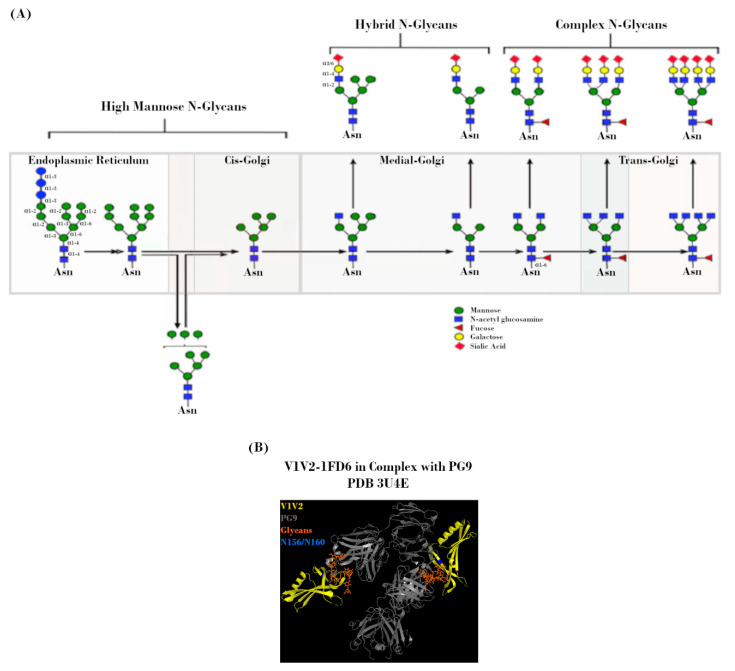
N-linked glycans on the HIV-1 envelope (Env). (**A**) Potential N-linked glycosylation site (PNGS) occupancy is initiated via the placement of a Glc_3_Man_9_GlcNAc_2_ residue, which is subsequently modified during transit through the endoplasmic reticulum (ER) and Golgi by a milieu of host enzymes to yield its final glycoform. Figure adapted from Jan et al., 2019 [49]. (**B**) Crystal structure (2.19 Å) of PG9 mAb in complex with V1V2 region from HIV-1. Regions corresponding to V1V2 (yellow), PG9 (grey), PNGS N156 and N160 (blue), and N-glycans (orange) are indicated. Protein Data Bank identifier (PDB ID) number is also indicated. Adapted from McLellan et al., 2011 [50].

The mechanism by which Env is processed prior to deposition on the viral surface is a key determinant in the makeup of the Env “glycan shield”. Shortly after SP-mediated delivery of the nascent Env polypeptide to the endoplasmic reticulum (ER) for processing, PNGS occupancy is initiated via the placement of a Glc_3_Man_9_GlcNAc_2_ residue at each site. During subsequent transit through the ER and Golgi, this precursor glycan is modified by a milieu of host enzymes to yield its final glycoform (Figure 3A). However, the accessibility of these enzymes is greatly affected by steric constraints related to PNGS occupancy, which is in turn dependent on viral strain, host cell type, and acquired mutations [33,35,36,37,38,39]. Due to the high level of overall PNGS occupancy, these steric constraints result in a glycan landscape that consists predominantly of high-mannose (immature/unprocessed Man_5-9_GlcNAc_2_) glycans, with a smaller proportion of more mature/highly-processed complex and hybrid glycans [39,40,41,42,43,44,45,46,47]. Even with such constraints, the total number of variables involved in this process leads to incredibly diverse glycan landscapes from the outset [41,46,48].

In much the same way that these glycans sterically hinder host glycosidases, they also confer resistance to nAbs [30,51,52]. Interactions between antibodies and these host-derived glycans are often low affinity, due to both the nature of such interactions as well as the fact that said glycans are immunologically “self” [53,54,55]. That is not to say that glycan-targeting bnAbs cannot exist, merely that glycans are often only one component of larger, non-linear epitopes [56]. Occupancy of specific PNGSs, such as N156 and N160, has been shown to be crucial for the formation of conformational epitopes recognized by bnAbs, such as PG9 and PG16 (Figure 3B) [57]. Additionally, one of the most common bnAb targets is a high-mannose patch centered around either N332 or N334, dependent on viral strain and antibody lineage [58,59,60,61]. This so-called glycan supersite is also the target of 2G12, which binds an epitope derived solely of glycans. Due to their dependence on higher-order conformation, attempts to elicit these bnAbs in the context of vaccination with recombinant Env constructs have not been successful. There is also evidence that glycans can modulate the composition of immune responses via interactions with antigen presenting cells. Glycans of the C2V3 region of Env have been shown in a mouse model to be capable of inducing a bias for unfavorable IgG1 antibody subtype and TH2 T cell responses [62].

Over the course of disease progression, selective pressure drives mutations that further aid in immune escape. These may manifest as altered PNGS locations, such as the N332 to N334 mutation that has been shown to mutate in both directions to avoid immune recognition [63]. They may also manifest as alterations in glycoforms, due to genetic or steric determinants [33,36,48,63,64,65,66,67,68,69,70]. Furthermore, single mutations that alter the occupancy or glycoform at any particular PNGS may have cascading effects on the processing of glycans at other PNGSs. Taken together, these factors contribute to a complex and constantly moving target for the immune system [71].

Despite this variation, a number of generalized structural and glycan motifs have been identified in HIV-1 isolates from varying stages of disease progression. In the vast majority of heterosexual infection events (>80%), a single transmitted/founder (T/F) strain is responsible for the establishment of infection [72,73,74,75,76]. For this reason, T/F strains are a prime target of interest for vaccines, microbicides, and pre- and post-exposure prophylactic measures. Cases involving multiple infecting strains are often tied to alternative transmission routes (such as intravenous drug use), or breakdown of mucosal barriers [75,77,78]. These T/F strains have been shown to possess a number of characteristics that separate them from chronic isolates and may partially explain why they are effective at establishing infection. For instance, T/F viruses from clades A, C, and D have been shown to possess shorter V1V2 regions and a reduced number of occupied PNGSs compared to chronic isolates [65,77,79,80,81,82,83,84]. Additionally, some clade B isolates have been shown to possess a shorter V5 region, as well as a V3 region that contains fewer occupied PNGSs and less positively charged residues [77,81,85,86,87,88,89,90]. As these variable regions are commonly targeted by nAbs, their shortening presents fewer potential targets for the immune system [91,92,93]. Due to their important roles in infection, many conserved sites (such as the CD4 and coreceptor binding sites) within these variable regions are rarely, if ever, directly occluded from immunological access via glycans. The lower PNGS occupancy observed in T/F viruses may reflect an adaptation to divert the immune response away from these crucial sites and onto residues in the vicinity of unoccupied PNGSs. Indeed, the earliest nAbs targeting T/F viruses are often specific for non-conserved residues within the variable regions of Env.

Wagh et al. recently utilized a computational model in order to characterize the relationship between the completeness of the glycan shield at transmission and the development of nAbs [51]. They found that a more complete glycan shield at transmission correlated with more rapid development of a broad nAb response. One possibility for this observation is that an increased initial PNGS occupancy represents fewer opportunities for escape. While not all escape mutations result in increased PNGS occupancy, we know that chronic isolates have more complete glycan shielding and a higher proportion of complex/hybrid glycans, suggesting a directionality to the process [6,68,77,94,95,96,97]. Additionally, as PNGS become occupied by primarily high-mannose glycans, the virus becomes more susceptible to capture and degradation by the C-type lectin DC-SIGN on dendritic cells and macrophages [49,98,99,100,101,102]. This leads to increased antigen presentation and therefore may increase the rate at which these rounds of escape and adaptation occur. Eventual increases in the proportions of complex/hybrid glycans decrease viral capture by DC-SIGN but are also temporally associated with the development of bnAbs [49,103]. However, virus that does end up captured via this route is protected from degradation by these more mature glycans, resulting in increased levels of CD4+ T cell transinfection and thus representing an additional viral route of infection free of bnAb interference.

It is clear that the contribution of glycosylation at all stages of HIV-1 infection is great, and therefore, understanding the mechanisms governing it is going to be crucial to the development of an effective vaccine. Recently, the Env SP has been implicated as an important determining factor in this process.

## 4. Role of the Signal Peptide

**Figure 4:**
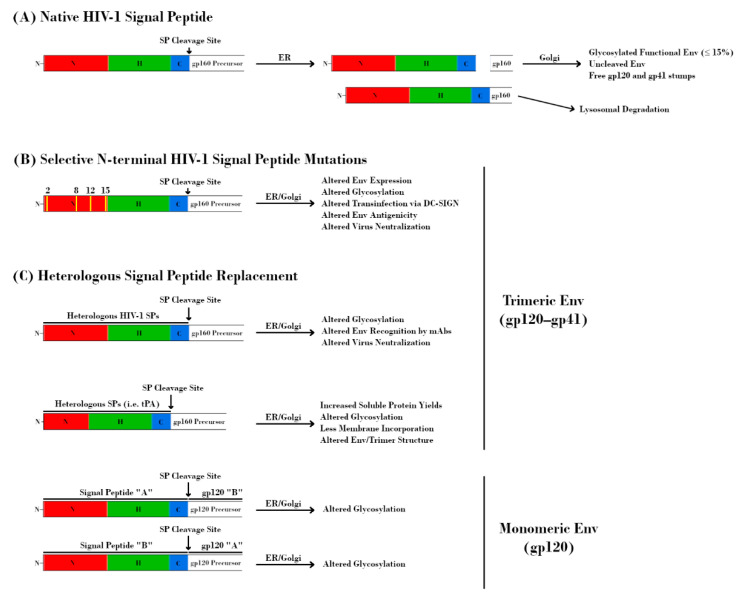
Effect of signal peptide (SP) modification/replacement on envelope (Env). (**A**) The HIV-1 SP is responsible for targeting the nascent glycoprotein 160 (gp160) precursor to the endoplasmic reticulum (ER) for processing. Uniquely, the SP remains attached to this protein throughout the ER and is cleaved prior to delivery of gp160 to the Golgi apparatus. Further retention of the SP results in lysosomal degradation of Env and serves as a quality control mechanism. (**B**) Mutations at specific residues within the N-terminal region of the HIV-1 SP result in alterations to Env expression, glycosylation, DC-SIGN-mediated transinfection, antigenicity, and neutralization. (**C**, top) Replacement of the native HIV-1 SP with heterologous HIV-1 SPs alters glycosylation, Env recognition by monoclonal antibodies (mAbs), and virus neutralization. Replacement with non-HIV-1 SPs is a common strategy for increasing immunogen yield. However, this approach may also have detrimental effects on Env glycosylation, structure, and antigenicity. The perceived advantage of increased soluble protein yields may be offset by a higher proportion of Env immunogens with under-processed glycans due to the absence of HIV-1 SP-mediated effects on processing. (**C**, bottom) The glycosylation profiles of HIV-1 glycoprotein 120 (gp120) proteins are dependent on their SPs. Transposition of SPs from HIV-1 species with different proportions of glycan types is sufficient to alter glycosylation profiles.

As with all membrane-bound and secreted proteins, the HIV-1 Env contains an N-terminal SP that is responsible for targeting the nascent polypeptide to the ER for processing. In general, SPs consist of an N-terminal hydrophilic positively charged region, a central hydrophobic region responsible for translocation to the ER membrane, and a slightly polar C-terminal region containing a cleavage site (Figure 1, bottom) [104]. The N-terminal region of the HIV-1 SP is relatively unique in that it is both longer and contains more positively charged residues than the average SP [105]. Additionally, the SP remains attached to gp160 until right before it reaches the Golgi, which is also unusual among SPs, most of which are cleaved co-translocationally (Figure 4A) [105,106,107,108]. 

The effects on Env processing due to these unique features may be explained by examining SPs from other systems. For instance, an increased positive charge in the N-terminal region is commonly seen in bacteria, especially Gram-positives [109]. Of note, many of these bacterial SPs are never cleaved from their mature proteins and perform additional functions related to membrane incorporation, secretion, and anchoring [110]. The HIV-1 SP is of course cleaved, as lack of SP cleavage prevents maturation of the protein, but it is quite possible that some of these functions overlap [111,112]. The N-terminal positive charge has also been shown in multiple systems to be crucial for SP orientation, which in turn affects additional SP functions such as facilitating membrane incorporation and regulating the timing of SP cleavage. Indeed, there is evidence that post-translocational cleavage of the HIV-1 SP is enabled via occlusion of the C-terminal cleavage site by SP itself [105,108,111,113]. The effect of increased SP length has also been studied extensively in other systems. The absolute length of SPs in each system varies greatly, but increased SP length is often associated with additional functions [114]. Of particular note are the SPs from human interleukin-15 and murine C4b-binding protein, which provide evidence for SP involvement in glycosylation efficiency and increased protein processing time, respectively [114]. These are but two examples of many but provide a precedent for the possibility that these functions, and potentially others, are being performed by the HIV-1 SP as well. Therefore, it is reasonable to speculate that the increased positive charge of the HIV-1 SP functions to facilitate membrane association of both the SP and nascent gp160. In the case of the SP, this helps to orient the SP in such a way that, in tandem with its increased length, occludes its cleavage site and prevents premature cleavage. Both the increased SP length and delayed cleavage provide more time for the processing of gp160, in the form of protein folding and enzymatic glycan modifications. The close membrane association also sterically influences glycan accessibility by glycosidases and increases the likelihood that functional Env trimers are incorporated into virions. A number of recent studies support these assertions and are discussed below [112,115,116,117].

The aforementioned differences in glycosylation patterns exhibited by T/F and chronic HIV-1 isolates were found to be correlated with mutations at specific sites within the N-terminal region of the SP. Broadly speaking, T/F strains were found to have a higher proportion of neutral and basic residues, which were lost in chronic isolates [82,115,118,119,120]. Chronic isolates were also shown to have increased basic residues within their hydrophobic region. This argues that the HIV-1 SP is under selective pressure during infection, and that the charge of these regions may affect membrane association and therefore alter glycosylation [82,118,121,122]. Several groups have utilized varying approaches in order to elucidate the role of these SP differences as they pertain to glycosylation and Env function. These include making complete SP swaps as well as more targeted changes, and utilize recombinant proteins, pseudoviruses, and infectious molecular clones (IMC).

The basic histidine residue at position 12 (H12) within acute/early viruses has been proposed to provide higher overall Env expression, which correlates with higher Env incorporation into virions and increased infectivity [82,115,123]. Using a targeted approach, Upadhyay et al. mutated basic residues within the N-terminal region of the Env SP and assessed their effects on Env functions and virus phenotype (Figure 4B) [121]. Three separate mutations at position 12 (H12R/Q/Y) resulted in decreased Env incorporation. Additionally, mutations at positions 8, 12, and 15 increased resistance to monoclonal antibodies (mAbs) targeting the V1V2 region of Env. Capture and transmission of virus via DC-SIGN was also affected by these mutations, with H12Q/Y exhibiting decreased capture and K2G, R15G, and H12/R/Q/Y exhibiting decreased transmission [121]. Importantly, these mutations were shown to alter the levels of α1-3/α1-2 mannose- and fucose-containing glycans on Env, as determined via lectin binding experiments and liquid chromatography–mass spectrometry (LC-MS/MS). These findings were observed in two separate HIV-1 strains (REJO and JRFL), demonstrating that they may be broadly applicable [121]. Further studies by this group assessed in more detail the contribution of glycans, and SP mutations affecting glycans, on DC-SIGN-mediated transinfection and interaction with antiviral lectins [49]. While the use of pseudoviruses (as opposed to IMCs) resulted in a few areas of divergence, these experiments further underscored the contribution of the SP in directing Env glycan composition. Of particular note was the observation that only SP-related mutations, not mutations directly affecting PNGS occupancy or overall gp120 conformation, had observable effects on DC-SIGN-dependent virus capture/transmission or lectin-mediated transmission inhibition [124,125]. Therefore, these findings argue that even modest changes in the SP at key residues can have widespread effects on the way that HIV-1 Env interacts with host cells and processes. Most recently, Upadhyay et al. observed that replacement of HIV-1 SPs with those from other HIV-1 isolates altered the proportions of oligomannose and complex glycans at the Env trimer apex (V1V2 and V3) and base (gp41 membrane proximal external region, MPER) (Figure 4C) [11]. These changes resulted in altered Env binding and virus neutralization by known mAbs, most prominently those targeting V2i and V2q epitopes. Furthermore, they demonstrated that these effects varied based on the Env backbone (CMU06, REJO, or SF162), as well as the host cell type (293T or PBMC) used for virus production. Utilizing recombinant gp120 proteins, Yolitz et al. also demonstrated that swapping SPs between HIV-1 strains affected the relative proportions of glycans such that they more closely resembled the strain from which the SP was derived (Figure 4C). The mass, glycosylation, interaction with DC-SIGN, and susceptibility to mAbs were all altered in these proteins [103]. Taken together, these studies underscore the important contribution of the HIV-1 SP with regards to glycosylation.

The crucial role of the HIV-1 SP in directing Env glycosylation no doubt has interesting implications for vaccine development as well. A major obstacle in the production of Env immunogens is the inherently low level of expression of these proteins under the control of the native HIV-1 SP. Heterologous SPs have long been used in lieu of HIV-1 SPs in recombinant vaccine constructs, as they have been shown to potentially increase immunogen yields in insect and mammalian expression systems (Figure 4C) [29,105,126,127,128,129]. Various heterologous SPs have been utilized in this way, with tissue plasminogen activator (tPA) being one of the most common, especially among the native-like trimers mentioned previously. Notably, this SP does not share the increased length and increased number of positively charged residues of the HIV-1 Env SP [122]. It is no doubt of importance to express usable quantities of immunogen, but the similarity of the immunogen to native HIV-1 Env is arguably more important. If modest changes to the SP can have such widespread effects on glycan composition and occupancy, then replacing it entirely is sure to complicate efforts to produce effective immunogens. Indeed, there is evidence of similar glycan composition on SOSIP.664 trimers despite being derived from early and late stages of infection, presumably due to the use of the same heterologous SP and in contrast to what is observed in HIV-1 strains [130]. The role of the HIV-1 Env SP in ensuring proper protein processing via its retention is also worth considering, as levels of functional Env are low (≤15%) even in the context of wild-type virus [131]. It is important to remember that the SP and Env protein have co-evolved. In these systems, there exists a balance between the amount of protein and the secretory machinery such that excess protein, even if it is functional, can be degraded if the system is overwhelmed. Heterologous SPs that increase total protein yield may be artificially selecting for products that mature more quickly due to co-translocational SP cleavage but do not present epitopes important for the generation of productive bnAbs [109]. Recent analyses of the PNGS occupancy of native-like trimers have indicated that, while there are generally high levels of occupancy, regions with many closely-spaced PNGSs (such as the V1V2 region) can sometimes have lower occupancy levels in these constructs [36,37,38,130]. This is likely due to a combination of an increased rate of processing due to codon optimization and a lack of the aforementioned quality control mechanisms. There has been a recent interest in creating membrane-bound native-like trimers, the thought being that the close proximity to the membrane may influence enzyme accessibility and therefore glycosylation. Interestingly, replacement of the SP has also been detrimental to these efforts, as it appears to decrease the amount of membrane incorporation compared to that of the native SP [122]. Therefore, it is possible that the benefits of using heterologous SPs may come at the cost of immunogen quality. Proponents of utilizing heterologous SPs may point to the replacement of the HIV-1 SP with that of the herpes simplex virus 1 (HSV-1) glycoprotein D (gD) SP in the RV144 trial as evidence that this practice is well tolerated. However, this replacement was done alongside an 11 amino acid deletion from the N-terminus of the gp120 immunogens, which was made to reduce errant immunogen dimerization and promote proper folding. Post-trial studies utilizing RV144 patient serum suggest that this deletion was also sufficient for enhanced antigenicity to C1, V2, and V1V2 epitopes [132,133]. It is then possible that utilizing native HIV-1 SPs may further increase the effectiveness of such an immunogen. However, it is worth noting that removal of these 11 amino acids also removes a region of Env shown to be important for SP retention and may therefore affect Env processing and glycosylation in much the same way its complete replacement does [111]. Further investigation focused on retaining native-like glycosylation and processing while also increasing protein yield is no doubt warranted.

## 5. RV144 and Beyond

Of the four HIV-1 vaccine approaches evaluated in clinical trials, only one—RV144—has demonstrated modest efficacy in preventing HIV-1 acquisition [133,134,135]. In this trial, two previously investigated but non-protective vaccines were combined (ALVAC-HIV and AIDSVAX B/E), and a prime-boost strategy was used. ALVAC-HIV (prime) was administered at 0, 4, 12, and 24 weeks and AIDSVAX B/E (boost) was administered at 12 and 24 weeks only. ALVAC-HIV utilizes a recombinant canarypox vector to express HIV-1 LAI Gag and Pol alongside monomeric 92TH023 gp120 linked to the transmembrane anchoring portion of LAI gp41. AIDSVAX B/E is a formulation of recombinant Env gp120 proteins from MN and A244 strains wherein the first 11 amino acids of Env and the native SPs are replaced with a 27 amino acid sequence from HSV-1 gD [134,136]. While the findings of this study are the subject of some controversy, it remains the best evidence that vaccine-mediated protection is a realistic and achievable goal [137,138,139,140,141]. For obvious reasons, the exact mechanism(s) by which this protective effect occurred continue to be a topic of much interest. The vaccine regimen induced HIV-specific humoral and cellular immune responses, and much emphasis has subsequently been placed on characterizing the contribution of antibodies targeting HIV-1 with regards to protection [135,142]. Of note, nnAbs containing Fc effector function and directed against conserved epitopes within the variable regions (V1V2 and V3) of Env were identified in the serum of RV144 patients [143,144]. Furthermore, the existence of these antibodies was correlated with decreased infection risk [145]. This humoral immune response was found to be robust, both immediately after the initial vaccine regimen and upon boost 6–8 years later, but in both cases, the response was short-lived [134,141]. Nonetheless, this trial highlighted the importance of antibody responses targeting V2 epitopes in vaccine-mediated protection. Based on the success of RV144, the HVTN100 vaccine trial was designed and carried out in South Africa [136,146]. This trial utilized a similar prime-boost strategy as RV144 but utilized immunogens based on clade C HIV, as this better reflected the HIV-1 strains circulating in that part of the world. These immunogens were also formulated with a more potent adjuvant than that utilized in RV144, and an additional boost at 12 months was included to test durability. Increased antibody titers and immune response durability were observed compared to RV144, and interim results were favorable enough to justify an additional trial intended to assess the effect of additional boosts on durability. Unfortunately, this trial—HVTN702—was discontinued in early 2020 due to lack of evidence that it had any efficacy in preventing HIV-1 infection. More specifically, an independent data and safety monitoring board (DSMB) examined HIV-1 infection rates after 60% of study participants had been participating for longer than 18 months and found that similar numbers of infections occurred in both the vaccine and placebo groups. While no additional vaccinations are being performed, the study participants are still being followed.

## 6. Broadly Neutralizing Antibodies and Current Vaccine Approaches

While not induced in the RV144 or HVTN100 vaccinees, bnAbs have been found in some chronically infected individuals. As of now, no bnAb has been identified that is capable of clearing infection in humans. The protective capacity of such antibodies has however been shown via passive and active immunization studies in mice and non-human primates [147,148,149,150,151,152,153,154,155,156,157,158,159,160,161,162,163,164,165,166,167,168]. Unfortunately, as in so many cases, promising results in animals do not necessarily translate to clinical efficacy in humans. Nonetheless, a number of bnAbs targeting the CD4 binding site and/or V3 glycan epitopes (such as VRC01 and PGT121) have been or are currently being evaluated as therapeutics in clinical trials [169]. The administration of heterologous bnAbs does, however, come with its own set of challenges, such as limited therapeutic duration due to relatively short half-lives. As such, a major focus of vaccines has been to induce the production of bnAbs within vaccinated individuals.

The methods utilized to elicit bnAbs via vaccination are extremely varied, both in their design and efficacy. However, they generally involve some combination of expression vectors, recombinant proteins, and genetic material. As mentioned above, both RV144 and HVTN100 utilized canarypox vectors to express monomeric Env immunogens alongside the administration of recombinant Env proteins [134,136]. Synthetic immunogens containing known bnAb epitopes coupled to immune-stimulating carriers have also been generated but have only resulted in nnAbs [170]. Another approach that is currently being investigated is the use of so-called mosaic immunogens which contain bioinformatically selected epitopes from multiple viral strains and proteins [171]. An advantage to this approach is that immunogens from many disparate geographical isolates can all be represented in one vaccine. This approach was initially designed to improve the breadth of cellular immune responses but has been shown to also induce cross-clade binding antibodies. A formulation utilizing an adenovirus serotype 26 (Ad26) vector expressing various Env/Gag/Pol immunogens in combination with recombinant glycoprotein 140 (gp140) has been shown to induce comparable immune responses in humans and rhesus macaques. A clinical trial utilizing this vaccine regimen, HV705, is currently underway in sub-Saharan Africa [171].

While elicitation of bnAbs is a rare event, even in the context of chronically infected individuals, attempts to induce protective bnAbs must take into account the possibility that the immunogen(s) being utilized are not accurate enough mimics of their native counterparts. The aforementioned native-like Env trimers represent efforts to address this possibility [9,10]. However, designing these trimers is likewise complicated. Iterative modifications intended to address various issues with stability, solubility, expression levels, and purification may very well compromise their effectiveness as immunogens. Furthermore, there has been recent interest in developing methods to mimic bnAb development during the course of infection, via engagement of naïve B cells producing germline bnAbs. These constructs have shown promise in proof-of-principle experiments utilizing transgenic animals [58,172,173]. However, these transgenic animal models represent optimal conditions for bnAb lineage development and therefore lack much of the complexity found in humans. The affinities of native-like trimers for the naïve B cell populations of interest will likely need to be specifically increased in order to overcome obstacles such as a lower frequency of B cell targets as well as the existence of other naïve B cell populations that may interfere. Regardless of the exact approach utilized, the contribution of steric and conformational determinants on epitope structure is increasingly being recognized as crucial to immunogen design, and therefore to the induction of bnAbs. This review highlights the importance of such determinants, especially those dictated by the unique features of the HIV-1 SP. Future attempts to elicit bnAbs would no doubt benefit from the use of native-like Env trimers generated using native HIV-1 SPs or SPs engineered to mimic them, given the important biosynthetic mechanisms (and, by extension, structural determinants such as glycosylation and higher-order protein structure) that they govern. The use of HIV-1 SPs will also likely aid in attempts to generate membrane-bound versions of these immunogens via increased membrane incorporation, which should further increase their similarity to their native counterparts. Finally, expressing these immunogens in cells that produce more similar glycosylation profiles to HIV-1 target cells could help as well.

## 7. Conclusions

Taken together, it is clear that the mechanisms by which HIV-1 evades the immune response—both in order to establish infection and in chronically infected patients—are extremely complex. A major contributor to this complexity is the existence of an array of N-linked glycans decorating the surface of the HIV-1 Env. The mechanisms governing the location, heterogeneity, and immunogenicity of these glycans are likewise complex, incorporating genetic, structural, and host-derived determinants. Regardless, evidence suggests that the HIV-1 SP plays an integral role in determining the composition of its glycan shield and must therefore be taken into consideration when designing immunogens for use in vaccination. Further investigation into the mechanisms by which this occurs will be important, both in the context of understanding HIV-1 biology and in order to inform HIV-1 vaccine design.

## Data Availability

No new data were created or analyzed in this study. Data sharing is not applicable to this article.
